# Exploration of silicon functions to integrate with biotic stress tolerance and crop improvement

**DOI:** 10.1186/s40659-021-00344-4

**Published:** 2021-07-08

**Authors:** Xiu-Peng Song, Krishan K. Verma, Dan-Dan Tian, Xiao-Qiu Zhang, Yong-Jian Liang, Xing Huang, Chang-Ning Li, Yang-Rui Li

**Affiliations:** 1 Sugarcane Research Institute, Guangxi Academy of Agricultural Sciences/Key Laboratory of Sugarcane Biotechnology and Genetic Improvement (Guangxi), Ministry of Agriculture and Rural Affairs/Guangxi Key Laboratory of Sugarcane Genetic Improvement, Nanning, 530007 Guangxi China; 2 Institute of Biotechnology, Guangxi Academy of Agricultural Sciences, Nanning, 530007 Guangxi China; 3 Guangxi South Subtropical Agricultural Science Research Institute, Chongzuo, 532200 Guangxi China

**Keywords:** Antioxidants, Biotic stress, Crop improvement, Physiology, Sustainable agriculture, Silicon

## Abstract

In the era of climate change, due to increased incidences of a wide range of various environmental stresses, especially biotic and abiotic stresses around the globe, the performance of plants can be affected by these stresses. After oxygen, silicon (Si) is the second most abundant element in the earth’s crust. It is not considered as an important element, but can be thought of as a multi-beneficial quasi-essential element for plants. This review on silicon presents an overview of the versatile role of this element in a variety of plants. Plants absorb silicon through roots from the rhizospheric soil in the form of silicic or monosilicic acid. Silicon plays a key metabolic function in living organisms due to its relative abundance in the atmosphere. Plants with higher content of silicon in shoot or root are very few prone to attack by pests, and exhibit increased stress resistance. However, the more remarkable impact of silicon is the decrease in the number of seed intensities/soil-borne and foliar diseases of major plant varieties that are infected by biotrophic, hemi-biotrophic and necrotrophic pathogens. The amelioration in disease symptoms are due to the effect of silicon on a some factors involved in providing host resistance namely, duration of incubation, size, shape and number of lesions. The formation of a mechanical barrier beneath the cuticle and in the cell walls by the polymerization of silicon was first proposed as to how this element decreases plant disease severity. The current understanding of how this element enhances resistance in plants subjected to biotic stress, the exact functions and mechanisms by which it modulates plant biology by potentiating the host defence mechanism needs to be studied using genomics, metabolomics and proteomics. The role of silicon in helping the plants in adaption to biotic stress has been discussed which will help to plan in a systematic way the development of more sustainable agriculture for food security and safety in the future.

## Background

The earth’s surface is covered with 28.8% (dry wt. basis) of silicon (Si) after oxygen, but the existence of Si in its pure form is extremely rare [1–3]. Silicon is found as quartz, feldspar, mica and clay minerals in the earth’s crust [2, 4, 5]. In biological systems, Si occurs in various forms of amorphous silica (SiO_2_nH_2_O) namely, as phytoliths and in silica rich plants [6, 7]. The biogenic share of silicon is about 1–3% of the total Si pool in the soil [8].

The numerous advantages of Si to crops are now widely acknowledged [3, 9–12]. The plant-available forms of Si may be limited [1, 13]. The plant biologists/physiologists recognized the significance of Si as early as the nineteenth century [14–16]. Silicon is now widely considered as a beneficial element [2, 17], but it is still considered non-essential for plant growth and development [18]. Lewin and Reimann [19] demonstrated that the Si played a major metabolic role in living organisms due to its relatively abundance in nature. According to Epstein [20], silicon is essential for plant growth, acts as a mechanical barrier and provides resistance against plant pathogens and herbivores, and as such drawn the more attention of its role in plant biology. Furthermore, [9] found that the majority of crops contain significant quantity of Si, implying that this is unlikely to be a result of stochastic element absorption, similar to how evolutionary mechanisms have evolved for uptake and accumulation of other nutritional elements.

Furthermore, Si uptake by plant roots can be adaptive in response to changing atmospheric variables namely, stress, whether abiotic or biotic [2, 3, 21, 22], and thus is not always necessary, but arguably important. While the importance of this feature to plants is still argued, considerable progress has been made in our understanding of Si uptake and accumulation in plants. Plants can be classified as hyper-accumulators, accumulators, passive accumulators and non-accumulating varieties. Furthermore, the benefits of application of liquid forms of Si have been demonstrated, especially during plant stress tolerance. Limited studies have been carried out whether Si plays a significant role in plant metabolic activities [2, 10, 22, 23].

The experiments showing the impact of Si on plant tolerance to environmental stresses [3, 24–28] have been carried out at physiological, molecular and ecological levels [12, 29–31]. The current research articles published on the mitigation of plant stress by Si demonstrates the interest in this area [3, 11, 22, 32, 33]. However, none of the researchers took into account the possibility of Si interacting with fundamental plant omics. Indeed, most studies have concentrated on species-specific and narrow aspects of Si–plant stress interactions. Some studies have contributed to our understanding of the larger effects of Si on plant growth and defence, including interactions with plant diseases, but the mechanisms underlying these effects are still unknown. The present review, briefly discusses the physiological and molecular basis of amelioration of biotic stress conditions in plants by silicon and the mechanisms involved.

### Biotic stress

In natural conditions, plants suffer from various types of stresses caused by living organisms like bacteria, viruses, fungi, parasites, significant and non-significant insects. Like livestock, plants also have a defence system, which provides tolerance against environmental stresses. On invasion by pathogens and herbivorous pests, plants make use of pre-existing physical, chemical and mechanical barriers to protect themselves. The plant defence functions are also activated upon attack by pest; plant protection functions as a unit to decrease negative responses of biotic stress (Table 1). The stress induced defence system is associated with complex interconnected pathways of signal transduction in which phytohormones namely, abscisic acid (ABA), ethylene (ETH), jasmonic acid (JA) and salicylic acid (SA) plays an important role [2, 13, 34–36]. The biotic stress may enhance in the coming years because of climate change. The costs linked with stress are potentially enormous, and the effects of the stress may have a great impact on sustainable agriculture and environmental systems [3].

**Table 1:** The adaptive mechanisms of silicon in crop plants against biotic stress

Stress/disease	Plant	Action	Source
Anthracnose	Tomato (*Solanum lycopersicum*)	Cuticle thickness and fruit firmness enhanced	[107]
Bacterial speck, Bacterial wilt, Fusarium crown and root rot	,,	Upgraded physiological, biochemical and molecular traits	[98, 106, 108, 109]
Early blight	,,	Improve biochemical and molecular aspects	[72]
Rice leaf folder	Rice (*Oryza sativa*)	Food/grain quality and conversion efficiencies decreased	[110]
Brown plant hopper	,,	Extraction of honeydew reduced	[111]
Fall armyworm	,,	Damage feeding preference as well as *S. frugiperda* larval survival	[112]
Sugarcane borer	,,	Feeding injury decreased and upregulated exposure to unfavorable climatic variables and natural enemies arising from decreased boring success	[113]
Blast, Brown spot, Grain discoloration, Leaf scald and Sheath blight	,,	Enhanced physical, biochemical and molecular activities	[41, 51, 67, 83, 114–120]
*Diatraeasaccharalis*	Sugarcane (*Saccharum* spp.)	Upgrade/maintain leaf cuticle thickening and leaf stomata crystals	[121]
Stalk borer	,,	Decreased % stalks and length bored	[122]
Brown rust	,,	Physical and biochemical	[123]
*Euschistusheros*	Soybean (*Glycine max*)	Upregulated non-preference and antibiosis resistances	[124]
Powdery mildew	Arabidopsis (*Arabidopsis thaliana)*	Physio-biochemical activities increased and/or balanced	[58, 75, 125]
Black sigatoka Fusarium wilt Root rot Xanthomonas wilt	Banana (*Musa* spp.)	,,	[126–129]
Powdery mildew	Barley (*Hordeum vulgare*)	Improve physiological performance	[130]
Angular leaf spot	Bean (*Phaseolus vulgaris*)	,,	[131]
Powdery mildew	Black gram (*Vigna mungo*)	Enhanced expression of genes	[132]
Dollar spot	Bentgrass (*Agrostis stolonifera*)	Improve physiological and biochemical characteristics	[90, 133]
Powdery mildew	Bitter gourd (*Momordica charantia*)	Enhanced biochemical activities	[134]
Anthracnose	Capsicum (*Capsicum annuum*)	Improve physiological and biochemical characteristics	[135]
Fruit decay	Cherry (*Prunus avium*)	Improve biochemical parameters	[136]
Fusarium root rot and Postharvest pink rot	Chinese cantaloupe	Improve physiological and biochemical characteristics	[137, 138]
Leaf rust and Root-knot Nematode	Coffee (*Coffea arabica*)	,,	[139, 140]
Anthracnose	Common bean (*Phaseolus vulgaris*)	Improve biochemical traits	[141, 142]
Fusarium wilt	Cotton (*Gossypium* spp.)	Improve physiological and biochemical characteristics	[143]
Crown and root rot, Fusarium wilt and Powdery mildew	Cucumber (*Cucumis sativus*)	,,	[37, 144–148]
Decay	Hami melons (*Cucumis melo*)	Improve biochemical activities	[149]
Downy mildew	Lettuce (*Lactuca sativa*)	Enhance physiological and biochemical activities	[150]
Bacterial fruit blotch and Powdery mildew	Melon (*Cucumis melo*)	Improve biochemical capacity	[151, 152]
Pink rot disease and Powdery mildew	Muskmelon (*Cucumis melo*)	Enhance physiological and biochemical activities	[146, 153]
Basal stem rot	Oil palm (*Elaeis guineensis*)	Balance physical characteristics	[154]
Brown spot	Pea (*Pisum sativum*)	Balance biochemical activities	[155]
Downy mildew	Pearl millet (*Pennisetum glaucum*)	Enhance physiological and biochemical activities	[156]
Fusarium patch and Gray leaf spot	Perennial ryegrass (*Lolium perenne*)	,,	[49, 157]
Dry rot	Potato (*Solanum tuberosum*)	,,	[137]
Powdery mildew	Pumpkin (*Cucurbita* spp.)	,,	[158]
Blast, leaf blast, leaf streak, powdery mildew and spot blotch	Wheat (*Triticum* spp.)	,,	[76, 159–162]

### Silicon mitigates biotic stress in plants: physical and mechanical barriers

Plants grown under normal conditions are exposed to environmental stresses such as biotic (caused by viral and bacterial pathogens or fungi and herbivores) and abiotic stresses (saline, high and low temperature, flooding, UV, wind, drought, metal toxicity, light and mineral deficiency or excess). Supplementing plants with Si have been shown to enhance plant tolerance to mammalian, arthropod, and molluscan herbivores, fungal and bacterial pathogens, viruses and nematodes [2, 21, 22, 26, 37, 38]. The physical defence induced by Si deposition in plant parts in the form of phytoliths (largely composed of SiO_2_) was one of the first theories proposed for studying stress tolerance to pests [39, 40]. Silicon translocated from the soil solution as monosilicic acid into plants. Monosilicic acid polymerizes to form phytoliths, which are accumulated within the plant in an irreversible manner [20, 29]. Deposition of phytoliths enhances plant immunity and physical resilience, and serves as a physical barrier to fungal penetration [41, 42]. Silicon deposition may also wear away the feeding mouthparts, or mandibles of insects [43], decrease plant digestibility for both insect and mammalian herbivores [21, 44, 45], and have an adverse effect on herbivores [46]. Importantly, plant tissue silicification may be induced more in those plants which are highly attacked by various organisms [42, 47]. Silicon also affects the plant metabolites associated with plant defence [10, 48, 49], such as chitinase (CHT), β-1,3-glucanase, phenylalanine ammonia-lyase (PAL), polyphenol oxidase (PPO), in a number of plant–pathogens such as necrotrophic, biotrophic and hemibiotrophic pathogens [37, 50, 51]. Silicon-induced increased production of flavonoids, peroxidases (PODs) and chitinase (CHT) in some necrotrophic pathogens have been reported [37, 52].

Current research has looked into the interactions between Si and plant defence signaling transduction pathways, specifically the main plant hormone signaling pathways. Plants develop a complex and unique blend of SA (generally linked with pathogens of (hemi)biotrophic), JA (generally linked with pathogens of necrotrophic and insect herbivores), and ETH (which is usually regarded as ‘fine-tuning' the JA defence action) in response to attack or infection [53, 54]. Plant hormone signaling has been shown to be important for Si-mediated plant tolerance to disease stress [50]. Ye et al. [55] demonstrated that the JA pathway needed for Si- induced insect herbivore tolerance uses JA-deficient rice mutants. Several other researchers demonstrated that the capacity of Si to induce JA-dependent defence functions, such as indirect insect herbivore attraction by changing the composition of herbivore-induced plant volatiles (HIPVs) generated during herbivore attack [56, 57]. Silicon increased SA-dependent defence genes in response to infection from biotrophic fungal pathogen, but induction of the SA pathway was not needed by Si to increase stress tolerance [58]. Van Bockhaven et al. [59] noted that the role of Si in increasing tolerance against necrotrophic fungal pathogen (*Cochliobolus miyabeanus*) was not dependent on the JA and SA pathways. Rather, they proposed that Si deactivated pathogen ethylene production by preventing the pathogen from hijacking the plant’s ETH mechanism. These findings indicated that the Si plays a significant role in multiple phytohormone signaling pathways to mitigate plant biotic stress [21, 28, 60].

Silicon can also help to overcome certain (hemi)biotrophic pathogens and the ability of some insects to suppress plant-induced defenses. When a plant detects a biotic threat, it triggers the defence phytohormone signaling pathway. Pathogen-associated (PAMPs), damage-associated (DAMPs) and herbivore-associated molecular patterns (HAMPs) are examples of conserved molecular patterns that differ depending on the plant disease [61, 62]. The identification of these molecules by pattern recognition receptors (PRRs), often in conjunction with identification of other pathogens/insects effector proteins, may activate a plant defense response (known as PAMP-triggered immunity [PTI] or effector-triggered immunity [ETI]) that is sufficient to induce plant stress [62, 63]. Though necrotrophic pathogens do not develop effector proteins [53], (hemi)biotrophic pathogens and herbivores do, effectively suppressing the plant immune response by suppressing PTI and ETI [64, 65]. Silicon accumulation in the plant apoplast is likely to prevent pathogen effectors from reaching their target sites, preventing the pathogen from inhibiting the plant defence response [58]. Silicon may be able to help in overcoming plant defence suppression, by enabling a complete defence response to be initiated when a biotic threat is present. One of the first cellular responses following identification of PAMPs or HAMPs is formation of reactive oxygen species (ROS), which helps in assessing biotic stress.

A common mechanism by which Si is proposed to function and mitigate biotic stress is ROS and enhanced antioxidant metabolism (a similar mechanism is involved in abiotic stress) [66]. Generation of ROS and increasing oxidative metabolism help to reduce oxidative damage to the plants [67, 68]. ROS generation and increased antioxidant metabolism have been linked to stress due to pathogen (bacterial and fungal) infection, as well as damage to the plant from chewing and sucking insects [67–69]. ROS may have a negative and direct effect on biotic stress [70]. However, ROS play a number of signaling actions in different defence signaling pathways with plant hormones, such as JA and SA [53, 70–72]. Additionally, ROS may stimulate plant defence genes, resulting in the accumulation of defence metabolic compounds such as phytoalexins and allelochemicals in the plants [73]. Van Bockhaven et al. [59] demonstrated that the primary plant metabolism, i.e. photorespiration and the development of ROS, play a significant role in the broad-spectrum impact of Si on disease mitigation. Silicon is needed for sustenance of life processes in diatoms (algal phytoplankton), including replication of DNA [74], and evaluating how Si affects the cellular metabolism in algae and other primitive plants could provide valuable insights into how Si functions and its mechanisms of action in angiosperms. The production of ROS as a by-product of fundamental life processes, as well as the implications of an association of Si with oxidation/antioxidant metabolism in numerous plant stress studies, indicate that this is a promising research avenue for determining fundamental role of Si in lower and higher plants.

In order to better understand how Si affects plant gene expression, researchers must combine transcriptomic approaches, i.e. microarrays with more focused assays, like real-time quantitative PCR (qRT-PCR). Fauteux et al. [75] observed that the defence genes of infected plants are upregulated and primary metabolism genes are downregulated. After the application of Si, the defence genes were less affected, and that there was no evidence to indicate that Si had an effect without pathogenic stress condition. Similarly, Chain et al. [76] and Van Bockhaven et al. [59] noticed that applying Si to plants almost completely eliminated the pathogen stress effects at the transcriptomics level (Tables 2, 3). In numerous studies, a higher Si content in the rhizospheric soil and growth medium has been shown to improve crop resistance to pest infection. With Si supplementation, white backed plant hoppers (*Sogatella frucifera*) have shown decreased feeding, decreased growth durability, decreased fecundity, and reduced population growth [77]. Furthermore, the foliar application of Si as calcium silicate (Ca_2_SiO_4_) to wheat (*Triticum* spp.), cotton (*Gossypium* spp.), sugarcane (*Saccharum* spp.), and cucumber (*Cucumis* spp.) enhanced white fly nymph mortality, resulting in substantial loss of crop production against normal plants [78]. The *Oryza sativa* plant roots with high Si content are resistant to rootknot nematode infection [79]. Silicon supplementation can also help rice plants resist attacks from green leaf hoppers, plant hoppers, and stem maggots [80]. Furthermore, leaf-eating caterpillars have been found to have a low preference for silicified plant parts [80]. Different approaches could be used to move Si-transporters from higher accumulator plants to plants lacking Si-transporters, thus providing protection against diseases.

**Table 2:** The role of defense-related enzymes regulated by silicon in biotic stress

Stress/disease	Plant	Antioxidants	Source
Anthracnose	Bean (*Phaseolus vulgaris*)	SOD, APX and GR	[141]
Powdery mildew, crown and root rot	Cucumber (*Cucumis sativus*)	POD, PPOs, CHT and POD	[37, 50]
Powdery mildew and pink rot	Melon (*Cucumis melo*)	POD, CHT, SOD, and β-1,3-glucanase	[149, 152]
Leaf spot	Pea (*Pisum sativum*)	CHT and β-1,3-glucanase	[155]
Blast, brown spot, sheath blight	Rice (*Oryza sativa*)	Glucanase, POD, PPOs, phenylalanine ammonia-lyase, SOD, CAT, APX, GR, lipoxygenase, Phenylalanine ammonia-lyases, CHT and β-1,3 glucanase	[51, 67, 116, 120, 163, 164]
Target spot	Soybean (*Glycine max*)	CHT, β-1-3-glucanases, phenylalanine ammonia-lyases, POD and PPOs	[165]
Blast	Wheat (*Triticum* spp.)	CHT and POD	[162]
Bacterial wilt and blight	Tomato (*Solanum lycopersicum*)	CAT, APX, SOD, GR POD and phenylalanine ammonia lyase	[72, 166]

**Table 3:** The role of genes upon the application of Si subjected to biotic stress/disease

Stress/disease	Plant	Functional annotation	Biological process	Function of Genes	Source
Rice blast	Rice (*Oryza sativa*)	β-1,3-Glucanase precursor, transport of heavy metal/detoxification protein domain-containing protein, pathogenic related transcriptional factor and ERF domain containing protein, precursor of peroxidase, resistance protein of bacterial blight and precursor of peroxidase	Defense	↑	[83]
		Stem rust tolerance protein of barley, family protein of disease resistance, HSP-20 domain containing protein, peroxidase, terpene synthase like protein and pathogenesis related protein type-I		↓	
		WRKY domain containing protein of DNA binding, transcriptional protein of trans-acting and R2R3 Myb protein (type-P)	Regulatory	↓	
Bacterial wilt	Tomatao (*Solanum lycopersicum*)	Stress responsive factor, pathogenesis related protein-1, β-glucanase, chitinase class II, peroxidase, phenylalanine ammonia lyase, Arabinogalactan protein and polygalacturonase inhibitor protein	Defense	↑	[98]
Rice blast	Rice (*Oryza sativa*)	Phosphoenolpyruvate carboxylase kinase, RNA-directed DNA polymerase (RT) domain containing protein, high pl α-glucosidase, oxalate oxidase like protein and P-type ATPase	Housekeeping	↑	[83]
		Family protein of putative cyclase, protein of transferase family, Dicyp-2 cyclophilin, DNA-directed RNA polymerase-2 and tyrosine decarboxylase I		↓	
		Cytochrome P450 monooxygenase	Photosynthetic	↓	
Bacterial wilt	Tomatao (*Solanum lycopersicum*)	Group of WRKY transcription factor-II, jasmonate and ethylene responsive factor-III and ferredoxin-I	Regulatory	↑	[98]

Arrow indicates increase and decrease activities

In Arabidopsis, it has recently been demonstrated that Si may protect plants from diseases through the SA-independent pathway. As a result, it was suggested that further work needs to be done on the SA-independent plant protection mechanisms, so that hybrid crops may be developed to cope with the changing environmental conditions [81]. Few studies have been conducted on the effect of Si on increased plant tolerance to insect herbivores. As a result, further research into the interactions of Si with the transcriptome of various varieties of plants whose Si uptake and accumulation ability varies (e.g. accumulators, non-accumulators) during attack by various types of insect herbivores (e.g. chewers, suckers) can provide useful insights into how Si alters plant gene expression in relation to insect stress.

### Impact of silicon on plants during favorable environmental conditions

It was earlier thought that Si had little or no impact on plant metabolism under controlled conditions [82]. In contrast, at present the effects of silicon on alleviation of biotic stresses are now well understood. Silicon has a profound effect on more basic metabolic processes [21, 22]. Recently, pot-based studies on the effect of Si on *Saccharum* spp. hybrid growth and its protection against an insect herbivore, showed substantial enhancement in plant growth and productivity [45]. Chain et al. [76] observed that the application of silicon to control plants of *Triticum aestivum* changed the regulation of 47 genes, while Brunings et al. [83] demonstrated that application of silicon to normal rice plants, changed the regulation of 221 genes, 28 of which were linked with defence and stress, and the rest were linked with primary metabolic mechanisms or whose functions were unknown. Van Bockhaven et al. [59] reported that Si changed the expression of genes involved in cell wall biosynthesis and glycolysis, as well as those of nitrogen and amino acid metabolism, and also affected the metabolism of defence hormones, namely ETH, JA and SA in rice plants.

Detmann et al. [84] showed the beneficial effects of silicon in rice plants. They concluded that the element enhanced photosynthetic capacity and, as a result, altered metabolism by stimulating amino acid remobilization, based on photosynthesis responses and transcriptomic and metabolomic profiling in paddy. According to Fleck et al. [85], Si significantly changed the root anatomy of normal grown crop plants, as well as the regulation of 265 genes, including a 25-fold increase of a particular protein-encoding gene that can play a key role in the perception of an unknown Si signal. Furthermore, Si has been shown to delay leaf senescence in both Si-accumulating and non-accumulating plant cultivars by activating the cytokinin pathway [86].

### Silicon mediated defense-related enzymes

Stress-related enzymes are strongly associated with disease tolerance, and Si has been observed to stimulate the activity of enzymes subjected to biotic stress [28, 72]. Many studies have assessed the impact of Si in disease tolerance by activating the activities of defence-related enzymes namely, chitinase (CHT), peroxidases (PODs), polyphenol oxidases (PPOs), β-1,3-glucanase, phenylalanine ammonia-lyase (PAL), superoxide dismutase (SOD), ascorbate peroxidase (APX), glutathione reductase (GR), catalase (CAT), lipoxygenase and glucanase [28, 72, 87] (Table 2). Application of Si could enhance the activity of POD and CHT, which play a significant role in biotic stress. POD is involved in cell-wall reinforcement, in the final stages of lignin biosynthesis, and in the cross-linking of cell wall proteins [88]. Defence-related enzymatic responses induced by Si can be associated with expression of genes related to enzyme synthesis [49]. The upregulated activities of antioxidative enzymes were monitored in cucumber, turfgrass and pea plants which were infected with powdery mildew, sheath blight and rust diseases, respectively [89–93]. Previous findings indicated that Si enhances SOD, CAT, APX, GR and POD content thereby, protecting the antioxidative metabolic processes [72, 94–96].

### Silicon modulated expression of genes

Housekeeping genes are essential for the proper functioning of cells and are expressed constitutively in all cells, regardless of the patho-physiological responses of these genes. Despite the fact that the expression of housekeeping genes is stable, some studies have found that they lose their stability when they are subjected to stress [22, 28, 83, 97]. According to Brunings et al. [83], Si application decreased the expression of essential housekeeping genes in rice under control conditions, but increased the expression of housekeeping genes to preserve cellular functions throughout pathogen infection. Silicon-mediated up-regulation of housekeeping genes such as actin (ACT), alpha-tubulin (TUB), and phosphoglycerate kinase (PGK) in *Ralstonia solanacearum*-infected in tomatoes [98]. According to Jarosch et al. [99], the actin cytoskeleton provided the basal resistance during infection in *R. solanacearum*. As a result, the host resistance was induced by the Si-dependent upregulation of actin in tomato (*Solanum lycopersicum*) plants [98]. Due to lack of a high-density Si transporter, tomato is classified as a low-level Si accumulator (about 0.2% dry weight) [100]. Furthermore, the application of Si in low-accumulating plants, i.e. *Solanum lycopersicum* [101], *Capsicum annuum* [102], and *Rosa* spp. [103] has resulted in overcoming stress resistance. Despite the fact that housekeeping genes have a constant expression level, variation in expression levels in response to Si treatment and pathogen infection can trigger the host plant's basal defence mechanism to protect it from the pathogen (Table 3).

Silicon is associated with the metabolic mechanisms of plant–pathogen interactions, triggering host plant defence genes via a sequence of physiological and biochemical reactions and signal transductions, as well as inducing disease resistance in plants [24, 58]. Silicon could play a role in the primary response, modulating the behavior of post-elicitation intracellular signaling pathways that control the expression of defence genes involved in cell wall structural modifications, hypersensitivity responses, synthesis of hormones, antimicrobial compound synthesis, and in formation of PR proteins [24]. To demonstrate the mechanism of protection of Si in various pathological systems, transcriptomic and proteomic experiments have been carried out [2, 21–23, 28, 98, 104].

The expression of genes encoding a novel proline-rich protein (PRP1) was increased under the induction of system acquired resistance in *Cucumis sativus* mediated by Si, which led to cell-wall reinforcement at the site of penetration of fungi into epidermal cells [105]. The expressions of CHI-II, GLU, PGIP, and POD, which are due to virulence factors released by the pathogen to inhibit host resistance and promote host invasion, were reduced by treatment with Si during pathogen interactions in tomato plants (*R. solanacearum*) [98]. Twenty six proteins were significantly altered by Si treatment in tomato plants, implying that Si-mediated disease resistance is linked to protein changes [106]. For example, Arabidopsis infected with the fungus, *Erysiphe cichoracearum* showed changes in the expression of about 4000 genes. The number and/or expression level of defence related genes enhanced in Si treated plants [75]. The expression of around 900 genes reacting to pathogen infection were modified in wheat plant leaves infected with *Blumeria graminis* f. sp. *tritici*, while the pathogen modified a few genes in silicon treated plants, implying that Si almost removed the stress due to pathogen invasion [76]. Brunings et al. [83] inoculated in the rice transcriptome, *Magnaporthe oryzae*, and riceblast fungus. Treatment of the plant with silicon appears to eradicate the effect of pathogen invasion on the transcriptome of host plants, rather than inducing resistance through transcriptional reprogramming of defence-related genes.

### Silicon as a sustainable alternative

Silicon has been shown to enhance crop resistance to a variety of biotic stresses and can be seen as an alternative to adaptive strategies [3, 10, 21, 22, 32]. Due to the special physical and chemical properties of Si have useful application in various sectors, including promising applications in the agri-sectors, they can easily enter into plant cells and affect the plant development by affecting their metabolism through diverse interactions, thereby triggering the potential to combat stress conditions. Thus, Si has the potential to be used as a fertilizer alone for specific crops and can be used to deliver herbicides and fertilizers in plants. The application of Si in agriculture may also lead to worldwide food security and safety by helping in the development of advanced varieties of crops with maximum yield. Silicon can provide green and eco-environment friendly alternatives to different synthetic fertilizers without environmental pollution. Simultaneously, the well-known positive impact on crop productivity and quality has a tremendous potential to enhance farmers’ profit margin through the utilization of the alternative approach.

## Conclusion and future prospects

Nowadays, there has been a lot of research which focuses on the role of Si in ameliorating plant tolerance to biotic stress, as well as in the regulation of signaling transduction pathways, and also in activating transcription factors in response to stress. Based on the present review, we concluded that Si increases plant resistance capacity to biotic stress, through a complex pathway associated with the plant defence system by activating transcription factors. In this review, we have discussed various aspects of Si and its regulatory functions during unfavorable conditions, and used key points from various relevant studies to explain how Si enhances stress resistance. While Si is associated with thousands of plant genes, it is not clear which other transcription factors and signaling proteins interact with Si to increase stress resistance. It will be very interesting to explore the role of Si signaling pathway, interactions with phytohormones, and crosstalk at the level of plant tissues, and at the cellular level to better understand how plants respond to environmental stresses, especially biotic stress. Overall, future research should concentrate on collecting more evidence to unravel the molecular mechanisms and the role of Si in plant tolerance to biotic stress, as well as the regulation of signal transduction pathways, and gene expressions associated in the biosynthesis of key compounds relevant to plant development.

## Data Availability

All the supporting data/findings are included in this article.

## Abbreviations

ABA: Abscisic acid
ACT: Actin
APX: Ascorbate peroxidase
CAT: Catalase
CHT: Chitinase
DAMPs: Damage-associated molecular patterns
ETH: Ethylene
ETI: Effector-triggered immunity
GR: Glutathione reducase
HAMPs: Herbivore-associated molecular patterns
HIPVs: Herbivore-induced plant volatiles
JA: Jasmonic acid
PAL: Phenylalanine ammonia-lyase
PAMP-PTI: PAMP-triggered immunity
PAMPs: Pathogen-associated molecular patterns
PGK: Phosphoglycerate kinase
POD: Peroxidases
PPOs: Polyphenol oxidases
PRP1: Proline rich protein
PRRs: Pattern recognition receptors
ROS: Reactive oxygen species
SA: Salicylic acid
Si: Silicon
SOD: Superoxide dismutase
TUB: Alpha-tubulin
